# Long-term effect of COVID-19 infection on hemodialysis patients: Should we follow hemodialysis patients more closely?

**DOI:** 10.1093/ckj/sfab265

**Published:** 2021-12-09

**Authors:** Atalay Demiray, Asiye Kanbay, Mehmet Kanbay

**Affiliations:** Department of Medicine, Koc University School of Medicine, Istanbul, Turkey; Department of Pulmonary Medicine, Istanbul Medicana Atasehir Hospital, Istanbul, Turkey; Department of Medicine, Division of Nephrology, Koc University School of Medicine, Istanbul, Turkey

**Keywords:** anti-SARS-CoV-2 antibodies, COVID-19, hemodialysis, mortality, outcomes

## Abstract

During the coronavirus disease 2019 (COVID-19) pandemic, hemodialysis patients constitute one of the most vulnerable patient populations as they have more significant comorbidities and need to visit healthcare settings frequently even under pandemic conditions. It was also largely demonstrated that hemodialysis patients have high mortality rates with severe to fatal disease due to COVID-19 during their initial hospitalization. Even though the functional decline and fatigue after severe infections are not novel entity, some long-term effects of COVID-19 has drawn attention with its prolonged effects even after discharge. A recent prospective, observational study of Carriazo et al provided the first evidence to compare long-term mortality rates of hemodialysis patients with and without COVID-19. Carriazo et al stated a hazard ratio of 3.00 for the mortality rates of hemodialysis patients over a one-year follow-up period after the COVID-19 diagnosis. They emphasized that the high mortality rates of hemodialysis patients with COVID-19 are not limited to the initial hospitalization period but also continue after discharge, especially the first three months. In the light of this study, it can be recommended that hemodialysis patients with COVID-19 should be monitored closely and continuously and hemodialysis patients should be prioritized for a vaccination against COVID-19 with close follow-up for their antibody levels.

ORIGINAL UNEDITED MANUSCRIPT

Carriazo et al. conducted a prospective, observational study to analyze the serological and clinical outcomes of 56 hemodialysis patients with coronavirus disease 2019 (COVID-19) for a year after the diagnosis (1). While current literature reported COVID-19 related mortality among hemodialysis patients during the initial hospitalization or one-month mortality after the diagnosis, this unique paper reported the outcomes of hemodialysis patients with prolonged follow-up. Carriazo et al. showed that high mortality rates of COVID-19 in hemodialysis patients are not limited to the initial hospitalization but also continue in the first year after the diagnosis. Among 56 hemodialysis patients with COVID-19, 20 (35.7%) died over 12 months with only 6 (11%) of them dying during the initial admission. Among 144 hemodialysis patients without COVID-19 infection, 21 (14.6%) died over 12 months. After one year of follow-up, they reported a hazard ratio for death for COVID-19 patients as 3.00 (1.62-5.53) with log-rank p=0.00023 (1). The findings of Carriazo et al made us question our approach in monitoring hemodialysis patients after COVID-19 and possible prolonged effects of COVID-19 on hemodialysis patients (Figure 1).

The number of patients with end-stage kidney disease (ESKD) is rising with the aging population and hemodialysis patients constitute a bigger burden in nephrology clinics (2, 3). The management and follow-up of hemodialysis patients have been a challenge for nephrologists especially during the COVID-19 pandemic (4). COVID-19 is a highly infectious viral disease with a reported case fatality rate of 15% in hemodialysis patients (5). Even though several drug regimens and protocols for COVID-19 have been prepared and worldwide vaccination against COVID-19 continues, the management of hemodialysis patients with COVID-19 appeared more challenging than other patients' populations due to some unknown aspects of COVID-19 (6). In the light of the present study, we can speculate two challenging aspects of COVID-19 infection that are needed to be elucidated are post COVID-19 syndrome and the rapidly declining nature of SARS-CoV-2 antibodies in hemodialysis patients.

Patients with chronic kidney disease (CKD), especially hemodialysis patients have several risk factors and comorbidities associated with severe or fatal COVID-19 (7-9). Hemodialysis patients are also vulnerable to COVID-19 because of impaired immune function, and frequent hospital visits as part of their life-sustaining therapy (9). A recent study with 576 hemodialysis patients showed in-hospital mortality (16.3%) due to COVID-19 and its determinants during hospitalization (10). However, it was unknown whether the effects of COVID-19 and high mortality rate are presented only acutely and limited to hospitalization or not. The study of Carriazo et al. demonstrated that high mortality rates of COVID-19 and its effects for hemodialysis patients could be continuing for one year, especially in the first three months after the diagnosis (1).

After COVID-19 discharge, functional limitation over a long period is very common due to post-infection fatigue. It is hard to distinguish this functional limitation and rehabilitation phase after discharge from post-COVID syndrome. However, several studies demonstrated prolonged effects of COVID-19, and some indicated post-COVID-19 syndrome (11). Even though the long-term effects of COVID-19 on CKD patients were not clearly demonstrated, the need for special rehabilitation programs and monitoring after discharge is recommended (12). In the light of the current study, we can speculate that the long-term consequences of COVID-19 on hemodialysis patients may not be just temporary post-infection fatigue, but a more severe entity needed to be elucidated to explain significantly high mortality rates even after discharge.

The second important issue of COVID-19 for hemodialysis patients is the rapid decline of SARS-CoV-2 antibodies compared to the general population. It is not novel that hemodialysis patients receiving immunosuppression therapies have suppressed immune response against infections and vaccinations. Additionally, immune responses and antibody formation in hemodialysis patients not on immunosuppressants were lower than in  the general population, so they are more prone to re-infection and faster loss of protection following vaccination. According to the study by Carriazo et al., only 30.6% of all COVID-19 survivor hemodialysis patients had anti-SARS-CoV-2 IgG after one-year follow-up. A decline in antibody levels against COVID-19 was observed between 6 and 12 months while some patients experienced re-infection and some never developed positive antibodies even after vaccination.

Besides comorbidities and altered immune responses, hemodialysis patients need to visit hemodialysis centers for their life-saving treatment three times a week and each visit can be a potential source for the transmission of COVID-19. Because these concerning aspects are specific to hemodialysis patients, it was broadly suggested that hemodialysis patients should be prioritized for vaccination and need to receive their COVID-19 vaccine as soon as possible (13). Hemodialysis patients should be followed more closely for their immune status, serological response, and possible re-infection. The study of Carriazo et al. pointed out that the presence of anti-SARS-CoV-2 IgG in hemodialysis patients decreased from 36/49 (73.4%) initially to 27/44 (61.3%) at 6 months, and 14/36 (38.8%) at 12 months (1). These findings made us question the immunity and antibody protection of hemodialysis patients with COVID-19 infection or vaccination. Therefore, possible COVID-19 re-infection should be kept in mind for hemodialysis patients even following vaccination.

There are some limitations of the study that could be addressed in the future. Carriazo et al. study presented a single-center experience with limited sample size. Multi-center data with bigger samples could validate and enlighten more about the long-term consequences of COVID-19 on hemodialysis patients. Even though the study presented clear data indicating high mortality rates even after initial hospitalization, more associations such as re-infections, hospitalizations due to other causes with longer follow-up periods should be assessed better for differentiating post-COVID syndrome (or effects) from general post-infection fatigue (or functional limitations).

In short, COVID-19 seems to have long-term consequences on hemodialysis patients with higher mortality rates not limited to the initial hospitalization period but also one year after the diagnosis. All infections tend to present more severely in hemodialysis patients due to comorbidities, drug regimens and, vulnerability than in other patient populations and COVID-19 is not an exception. COVID-19 is not the first pandemic and it will not be the last one. Future pandemics will also be threats to the well-being of hemodialysis patients. It should be kept in mind that hemodialysis patients always constitute a more vulnerable population in pandemics, epidemics, and infectious disease in general. Considering an increasing number of hemodialysis patients worldwide, their management in the case of COVID-19 and possible future infections should be well-tailored and closely followed.

## ACKNOWLEDGEMENTS

MK gratefully acknowledge use of the services and facilities of the Koc University Research Center for Translational Medicine (KUTTAM), funded by the Presidency of Turkey, Presidency of Strategy and Budget. The content is solely the responsibility of the authors and does not necessarily represent the official views of the Presidency of Strategy and Budget.

## FUNDING

This study was not funded by any grant.

## CONFLICT OF INTEREST STATEMENT

MK is member of the CKJ editorial board. The other authors declare that they have no conflict of interest.

## ETHICAL APPROVAL

This article does not contain any studies with human participants or animals performed by any of the authors.

ORIGINAL UNEDITED MANUSCRIPT

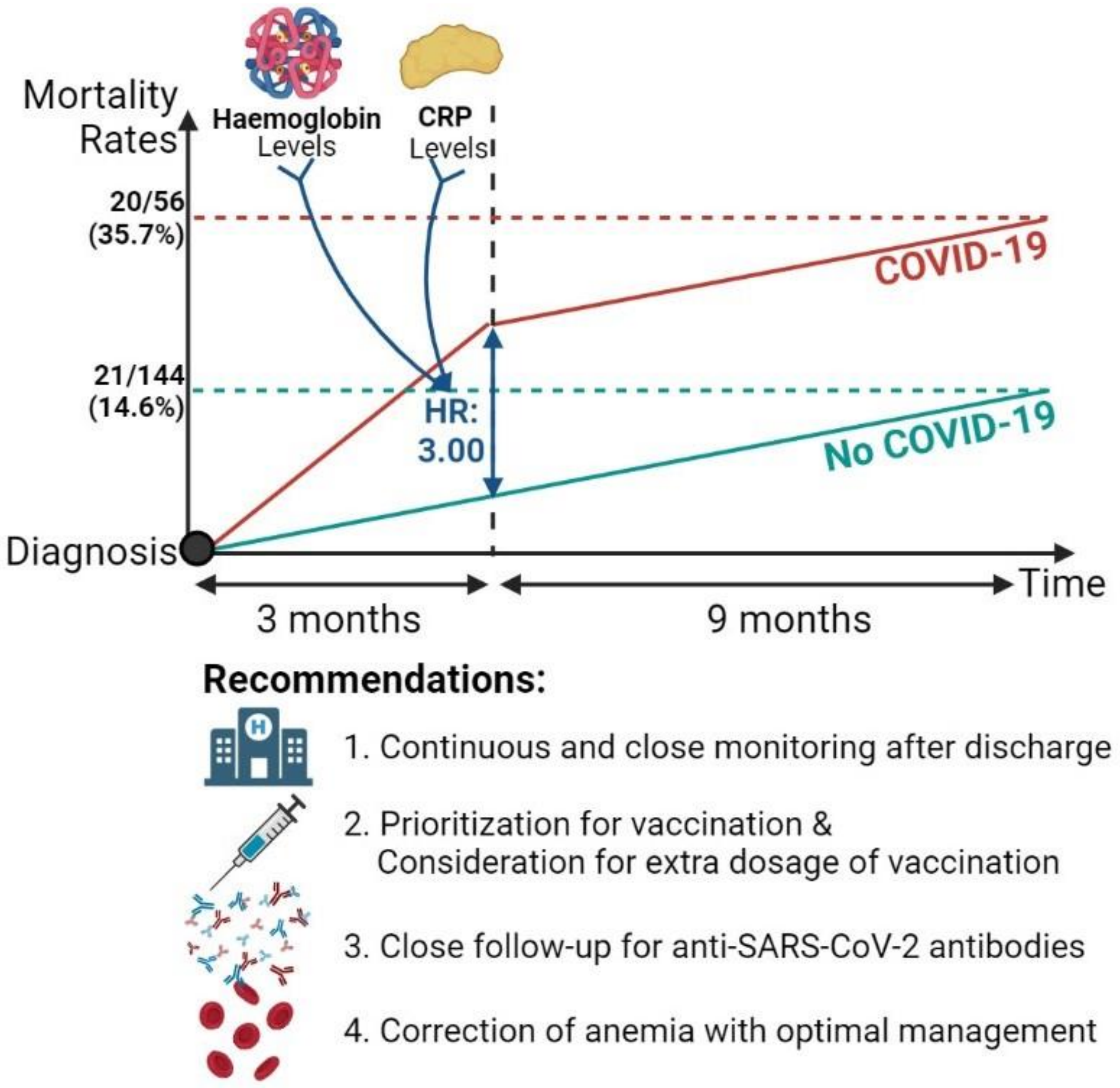

**FIGURE 1:** The simplified graph for mortality rates of hemodialysis patients with and without COVID-19 and recommendations for hemodialysis patients with COVID-19.

ORIGINAL UNEDITED MANUSCRIPT
